# GNG4, as a potential predictor of prognosis, is correlated with immune infiltrates in colon adenocarcinoma

**DOI:** 10.1111/jcmm.17847

**Published:** 2023-07-13

**Authors:** Juan Wang, Yanshuang Wang, Jiaming Zhou, Mengmeng Cai, Peng Guo, Tongde Du, Hui Zhang

**Affiliations:** ^1^ Department of Oncology Dushu Lake Hospital Affiliated to Soochow University Suzhou China; ^2^ State Key Laboratory of Proteomics, National Center for Protein Sciences (Beijing) Beijing Institute of Lifeomics Beijing China; ^3^ Department of Endoscopy Cancer Hospital of the University of Chinese Academy of Sciences (Zhejiang Cancer Hospital), Institute of Cancer and Basic Medicine (IBMC), Chinese Academy of Sciences Hangzhou China; ^4^ Suzhou Institute of Systems Medicine Suzhou China

**Keywords:** colon cancer, GNG4, immune infiltration, immunotherapy, tumour microenvironment

## Abstract

The tumour microenvironment (TME) and immunosuppression play an important role in colon cancer (CC) metastasis, which seriously affects the prognosis of CC. G protein subunit gamma 4 (GNG4) has been shown to participate in tumour progression and the tumour mutation burden (TMB) in colorectal cancer. However, the effect of GNG4 on the CC TME and immunology remains elusive. Weighted gene coexpression network analysis (WGCNA) was employed for screening aberrantly expressed genes associated with the immune score, and *GNG4* was then selected through prognostic and immune correlation analysis. Based on RNA sequencing data obtained from the TCGA and GEO databases, the expression pattern and immune characteristics of *GNG4* were comprehensively examined using a pan‐cancer analysis. Upregulation of *GNG4* was linked to an adverse prognosis and immune inhibitory phenotype in CC. Pan‐cancer analysis demonstrated higher *GNG4* expression in tumours than in paired normal tissue in human cancers. *GNG4* expression was closely related to prognosis, TMB, immune checkpoints (ICPs), microsatellite instability (MSI) and neoantigens. GNG4 promoted CC cell proliferation, migration and invasion and participated in immune regulation in the TME. Significantly, *GNG4* expression was found to negatively correlate with tumour‐infiltrating immune cells, ICP, TMB and MSI in CC. *GNG4* expression predicted the immunotherapy response in the IMvigor210 cohort, suggesting that *GNG4* could be used as a potential biomarker in CC for prognostication and immunology. Moreover, the expression of *GNG4* predicted the immunotherapy response of ICB in CC.

## 1 INTRODUCTION

Colorectal cancer (CRC), with the third highest morbidity and mortality rates, is one of the most common malignant tumours in the world.^1^ Twenty percent of patients have metastatic CRC when first diagnosed with CRC, and another 25% of patients with local tumours will eventually develop metastasis. Fewer than 20% of patients with metastatic CRC survive for more than 5 years.^2^

Cancer immunotherapy has shown promise in the treatment of recurrent or metastatic cancer.^3^ ICB has demonstrated durable responses and long‐lasting clinical benefits for a wide range of solid tumour types. Pembrolizumab and nivolumab are efficient for treatment of metastatic CRC with deficient mismatch repair (MMR) or high microsatellite instability (MSI‐H), for which accelerated FDA approval have been granted for two promising programmed cell death 1 (PD1)‐blocking antibodies^4^ in CRC. However, the limited response of treatment in patients suggests the importance of effective markers for immunotherapy.^5^

Currently, multiple potential factors have been identified, including the tumour mutation burden (TMB),^6^ MSI status,^7^ neoepitope load,^8^ PD‐L1 level,^9^ CD8 ^+^ T‐cell density,^10^ interferon‐γ gene signature,^11^ and MHC and T‐cell receptor repertoire.^12^ However, those biomarkers lack extensive validation and adequate data support. Given the severe side effects and substantial economic burden of cancer treatments, novel and versatile biomarkers that can predict the ICB response are urgent.^13^

Herein, a comprehensive analysis using public databases of colon cancer (CC) was conducted, in which GNG4 was identified as an immunotherapy marker associated with prognosis. GNG4, one of the 14 γ subunits of human G proteins, is crucial in guanosine triphosphatase (GTPase) activity, G protein–effector interactions and guanosine diphosphate (GDP) synthesis. Notably, hypermethylated *GNG4* was found in bladder cancer and glioblastoma.^14^, ^15^ Furthermore, high expression of GNG4 has also been associated with the progression of CRC^16^ and a variety of malignant phenotypes of lung adenocarcinoma.^17^ GNG4 was demonstrated to be the key element of the CRC TMB, which is essential for immune checkpoint inhibitor (ICI) therapy of CRC.^18^ However, the carcinogenesis and immunoregulatory role of GNG4 in the CC microenvironment requires further investigation.

## 2 METHODS

### 2.1 Differentially expressed gene (DEG) analysis in CC

RNA expression profiles (RNA‐Seq2 level 3 data) were obtained on an Illumina HiSeq 2000 from the TCGA database (https://portal. gdc.cancer.gov/), and contained 480 CC samples and 41 normal colon tissue samples. From the GEO database (https://www.ncbi. nlm.nih.gov/gds), the GSE39582 data set containing 566 CC samples and 19 normal colon tissue samples was downloaded. The ‘edgeR’ and ‘limma’ packages were used to screen DEGs from the TCGA and GEO databases, based on |log FC| (>1) and adjusted *p* (<0.01) value, respectively.

### 2.2 Immune score evaluation, WGCNA construction and gene set enrichment analysis (GSEA)

The tumour purity and immune score for 566 CC samples in the GSE39582 data set were calculated using the ‘estimate’ package in R based on their gene expression matrix. Then, the DEG matrix was further analysed by WGCNA. A correlation between the gene modules and an immune score greater than 0.3 was used to identify immune‐related gene sets. Finally, *GNG4* as a potential predictor of prognosis correlating with immune infiltrates was determined through the GEPIA and Timer databases.

To investigate the potential mechanism, the RNA expression profiles were divided into two groups according to the *GNG4* median value. GSEA was applied using the R package ‘clusterProfiler’ based on the DEGs between the two *GNG4* expression groups. The gene sets of c5.go.bp.v7.4.symbols.gmt were obtained from GSEA (http://www.gsea‐msigdb.org/gsea/index.jsp).

### 2.3 Pan‐cancer analysis

The TIMER2 database (http://timer.comp‐genomics.org/) was used to compare *GNG4* expression in human cancers with that in paired normal tissue.^19^ The GEPIA 2 database (http://gepia2.cancer‐pku.cn/#index) was employed for analysing the prognostic value of *GNG4* and the tumour node metastasis (TNM) stage in human cancers. Associations between *GNG4* expression and various immune signatures (immunoinhibitor, immunostimulator, MHC molecule, chemokine and receptor) were obtained from the TISIDB database (http://cis.hku.hk/TISIDB/index.php).^20^ ICP data were downloaded from the TCGA database. The differences in the tumour‐infiltrating fractions of 28 human immune cell phenotypes were evaluated by the single‐sample ssGSEA based on the ‘gsva’ package (v1.40.1) in R. The relationship between *GNG4* expression and TMB, mutant‐allele tumour heterogeneity (MASH), as well as MSI were studied using the SangerBox website (http:// sangerbox.com/Tool).

### 2.4 *GNG4* methylation analysis

The methylation data for the *GNG4* promoter were downloaded from the TCGA database. CpG island methylation data were visualized by the MEXPRESS database (https://mexpress.be/).^21^

### 2.5 *GNG4* expression and copy number variation (CNV) and drug sensitivity exploration

CNV data of CC patients were also obtained from the SangerBox website. The CellMiner (https://discover.nci.nih.gov/cellminer/) web tool was used to assess the relationship between *GNG4* expression and pharmacological data in the NCI‐60 cell line set.^22^

### 2.6 Cell culture

Human CC cells SW480, HCT116, DLD1 and RKO and the human colon epithelial cells NCM460 and HCoEpiC were obtained from the Shanghai Cell Bank of the Chinese Academy of Science and cultured in DMEM medium with 10% foetal bovine serum (FBS). All of the cell types were incubated in a humidified atmosphere with 5% CO_2_ at 37°C. The cell medium was changed every 2 days based on this culture environment.

### 2.7 Quantitative real‐time PCR (RT‐qPCR)

Total RNA from cells was extracted using an EZ‐10 DNAaway RNA Mini‐prep kit (Sangon Biotech Co., Ltd.). After the concentration and quality of RNA at 260/280 nm absorbance was determined, reverse transcription was performed by using PrimerScript RT Master mix (Takara Biotechnology Co., Ltd.). All of the PCR primers were obtained from Sangon Biotechnology. An ABI 7300 PCR system (Applied Biosystem) was used to perform the quantitative PCR reaction with SYBR Green Master Mix (Thermo Fisher Scientific). The primer sequences were as follows: *GNG4* (forward primer, 5′‐GCATCTCCCAAGCCAGGAAAGC‐3′ and reverse primer, 5′‐ GCAGGCACTGGAATGATGAGAGG‐3′). Relative expression was normalized to *GAPDH* as an internal control and calculated using 2^−△△CT^.

### 2.8 Cell viability testing

A cell counting kit‐8 (CCK‐8) was used to evaluate the cell survival. Cells were seeded in 96‐well plates for different times (24 h, 48 h and 72 h). Then, 90 μL of DMEM and 10 μL of CCK‐8 working solution were added to each well. The cells were incubated at 37°C for 1 h and detected at 450 nm on a SpectraMax spectrophotometer (Molecular Devices) to calculate the optical density (OD) values.

### 2.9 Transwell assay

For the Transwell assay, 5 × 10^4^ cells/well were resuspended in 200 μL of serum‐free medium in the upper chamber and 600 μL of medium supplemented with 10% FBS were filled in the lower chamber (8‐μm pore size, Coster, Corning, USA). After incubation for 48 h (migration assay) or 72 h (invasion assay) in a humidified atmosphere (5% CO_2_ at 37°C), the cells in the parietal chamber were removed. Then the cells on the submucosal surface were immobilized in 4% paraformaldehyde for 30 min and stained with a crystal violet solution. The number of migrated cells in the four random regions of each membrane layer was counted under the microscope.

### 2.10 *GNG4* expression and immune subtypes, molecular subtypes and ICB

The TISIDB database was used to explore the correlations between *GNG4* expression and immune subtypes or molecular subtypes of other cancers, in which a *p* value <0.05 was set as the satisfying criteria. The ‘IMvigor210CoreBiologies’ (version 1.0.0) package in the R software containing clinical data of anti‐PD1 therapy in patients with advanced uroepithelial carcinoma was used to evaluate the predictive effect of *GNG4* on immunotherapy.^23^

### 2.11 Lentivirus packaging and infection

The *GNG4* shRNA sequence was cloned into the pLKO‐puro vector to generate the lentiviral shRNA constructs against human *GNG4*. pLKO.1, pVSVG, pREV and pGAG were cotransfected into HEK293T cells for 24 h, and cell culture media were collected. The viruses were used to infect HCT116 CC cells in the presence of polybrene. Forty‐eight hours later, HCT116 cells were cultured in medium containing puromycin for the selection of stable cells. The cells stably knocking down were identified and verified by Western blotting.

### 2.12 Human tumour xenograft models

BALB/c nude mice (6‐week old, 18.0 ± 2.0 g) were purchased from Beijing Vital River Laboratory and were randomly divided into indicated groups. The mice in the groups were subcutaneously injected with the indicated cells stably expressing the indicated shRNA or control. Tumour size was measured every 3 days by Vernier calliper and converted to TV according to the following formula: TV (mm3) = (a × b2)/2, where a and b are the largest and smallest diameters, respectively. All animals were killed 22 days after measurement, and the transplanted tumours were removed, weighed and fixed for further study. The animal experimental protocols were approved by the Animal Care and Use Committee.

### 2.13 Statistical analysis

All of the statistical analyses were performed using SPSS software (version 25.0) or R software (version 4.0.3). Spearman's coefficients was used to evaluate the correlation between *GNG4* expression and variables. Statistical significance was determined as: NS, not significant; *p < 0.05; **p ≤ 0.01; ***p ≤ 0.001.

## 3 RESULTS

### 3.1 *GNG4* is an immunogenic‐related gene affecting the prognosis of CC patients as screened by WGCNA

**FIGURE 1:**
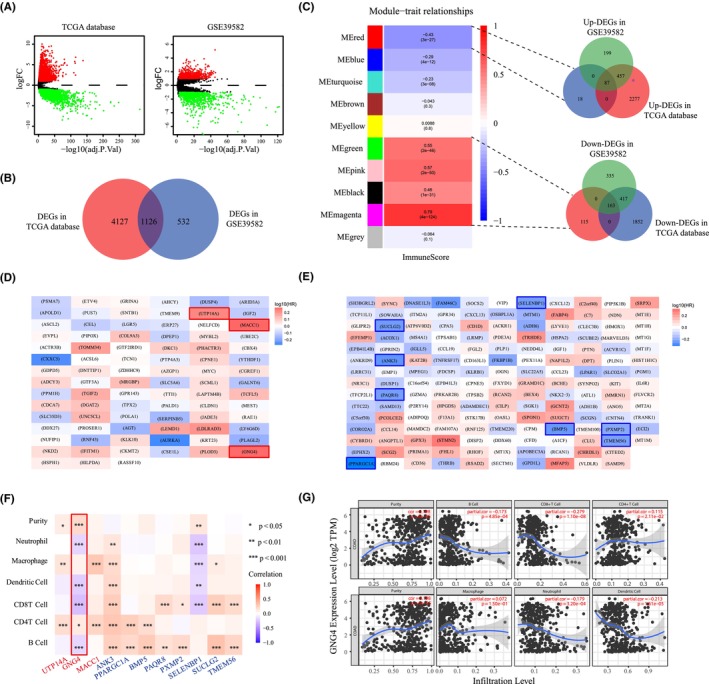
WGCNA analysis revealed *GNG4* was associated with immune infiltration in colon cancer. (A) Common DEGs between the normal and tumour groups in the TCGA‐COAD and the GSE39582 data set based on the criteria of |logFC| < 1 and adjust *p* value <0.01. (B) Intersection of DEGs in the TCGA‐COAD and GSE39582 data sets. (C) Identification of immune‐related modules by WGCNA analysis. (D) Heat map of the prognostic analysis of upregulated and negative immune‐associated DEGs in the GEPIA2 database (genes in red boxes represent high expression with a poor prognosis). (E) Heat map of the prognostic analysis of downregulation and positive immune‐associated DEGs in the GEPIA2 database (blue boxes represent high expression with better prognosis). (F) Immune cell correlation analysis of 11 candidate genes in the Timer database (upregulation in red and downregulation in blue). (G) Correlation analysis between GNG4 and immune cells was performed in the Timer database.

As shown in the volcano plots in Figure 1A, dysregulated genes between normal and tumour groups were identified based on the criteria of |logFC| < 1 and an adjusted *p* value <0.01. Then, 5253 DEGs (2821 upregulated genes and 2432 downregulated genes) were found in the TCGA‐COAD data set and 1673 DEGs (743 upregulated genes and 915 downregulated genes) from the GSE39582 data set. The consistently dysregulated genes were further selected by the intersection of the data sets (Figure 1B), and the final 1126 DEGs were used for subsequent WGCNA analysis.

We hypothesized that gene modules with high expression and a negative correlation with the immune score or gene modules with low expression and a positive correlation with the immune score in tumours could serve as potential therapeutic targets. Ultimately, we identified 87 genes negatively associated with the immune score and 163 genes positively associated with the immune score (Figure 1C). We identified three oncogenic genes that were positively correlated with prognosis, highly expressed and negatively correlated with immune invasion (Figure 1D) from the GEPIA2 database. Similarly, as shown in Figure 1E, eight suppressor oncogenes were screened, which were negatively correlated with prognosis, with low expression in tumours and positively correlated with immune invasion. *GNG4* was further identified by immune cell correlation analysis in the Timer database (Figure 1F). *GNG4* was highly expressed in CC. High *GNG4* expression was detrimental to patients, including a poorer prognosis, lower immune score and fewer immune cells (Figure 1F,G), especially CD8^+^ T cells.

### 3.2 GSEA of *GNG4*

**FIGURE 2:**
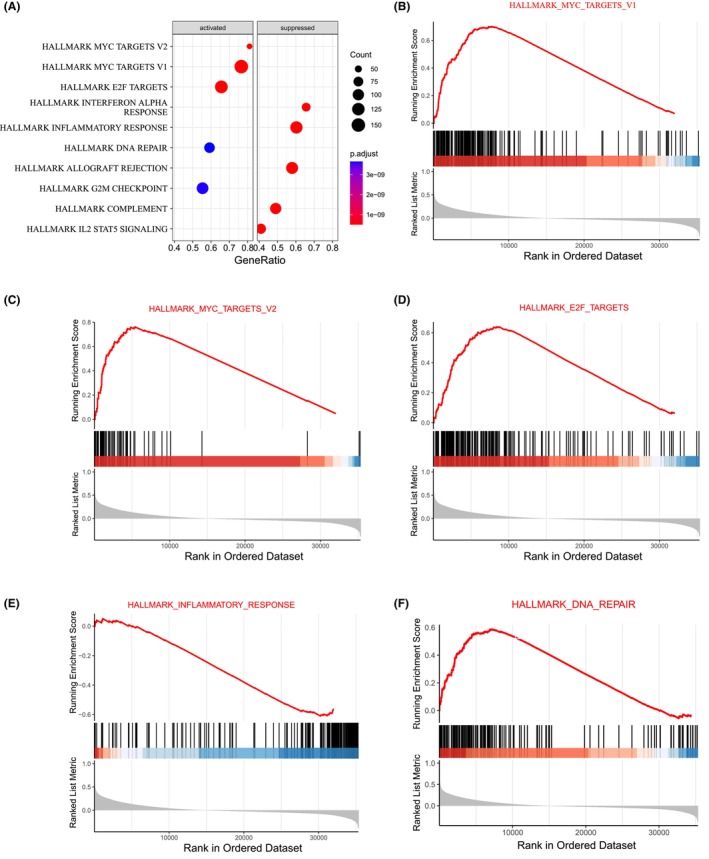
GSEA enrichment analysis of GNG4‐related signalling pathways. (A) Scatter plot of the top 10 pathways most associated with GNG4. (B) MYC targets; (C) E2F targets; (D) interferon α response; (E) inflammatory response; (F) and DNA repair (Figure 2B–F).

To explore the possible mechanism of GNG4, RNA‐seq data were grouped according to the *GNG4* median value in the TCGA database, and the DEGs further underwent GSEA (Figure 2A). The results of KEGG enrichment analysis revealed immune‐related signalling pathways, including MYC targets (Figure 2B,C), E2F targets (Figure 2D), inflammatory response (Figure 2E) and DNA repair (Figure 2F).

### 3.3 *GNG4* is abnormally expressed in human cancers and associated with a poor clinical prognosis

**FIGURE 3:**
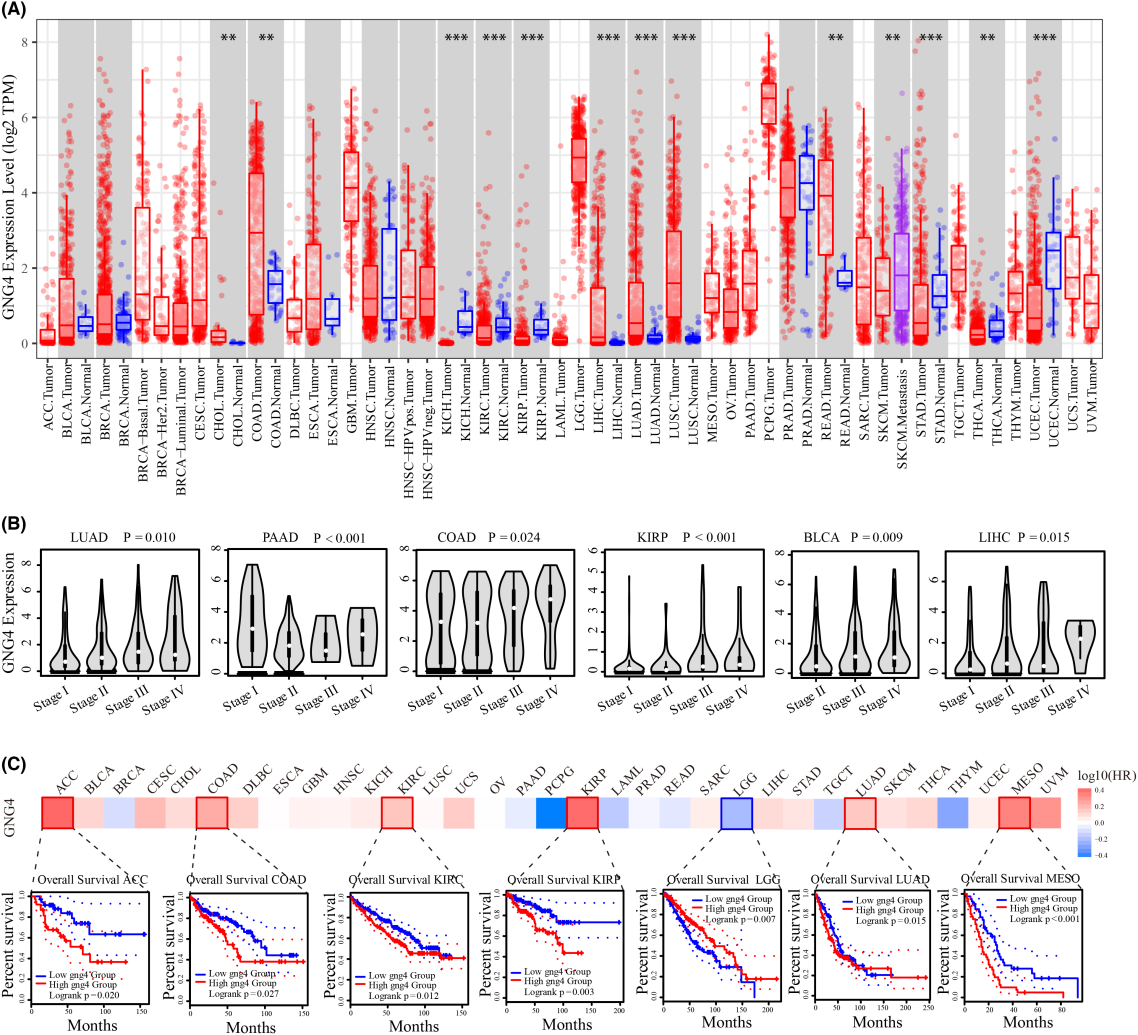
Correlation between *GNG4* expression and clinicopathologic data across cancer types. (A) Expression of *GNG4* mRNA between cancer and adjacent normal tissue. (B) Correlation between *GNG4* expression level and TNM stage. (C) Correlation between *GNG4* expression level and overall survival.

A pan‐cancer analysis of *GNG4* using the Timer database was performed. Of the 17 cancers with normal matched tissue, *GNG4* mRNA expression was upregulated in human cancers such as CHOL, COAD, LIHC, LUAD, LUSC and READ, and downregulated in cancers such as KICH, KIRC, KIRP, STAD, THCA and UCEC. As shown in Figure 3A, *GNG4* expression presented no significant difference in BLCA, BRCA, ESCA, HNSC or PRAD. As shown in Figure 3B, *GNG4* expression increased in patients with a high TNM stage in LUAD, PAAD, COAD, KIRP, BLCA and LIHC. The prognostic value of *GNG4* expression was further analysed. High *GNG4* expression was associated with a poorer prognosis and lower overall survival (OS) in patients with ACC (*p* = 0.020), COAD (*p* = 0.027), KIRC (*p* = 0.012), KIRP (*p* = 0.007), LUAD (*p* = 0.015) and MESO (*p* < 0.001). However, KIRP patients with high *GNG4* expression had a better prognosis (Figure 3C). The above data indicated that *GNG4* is an oncogene that plays a role in most cancers.

### 3.4 Pan‐cancer immunological correlation of GNG4

**FIGURE 4:**
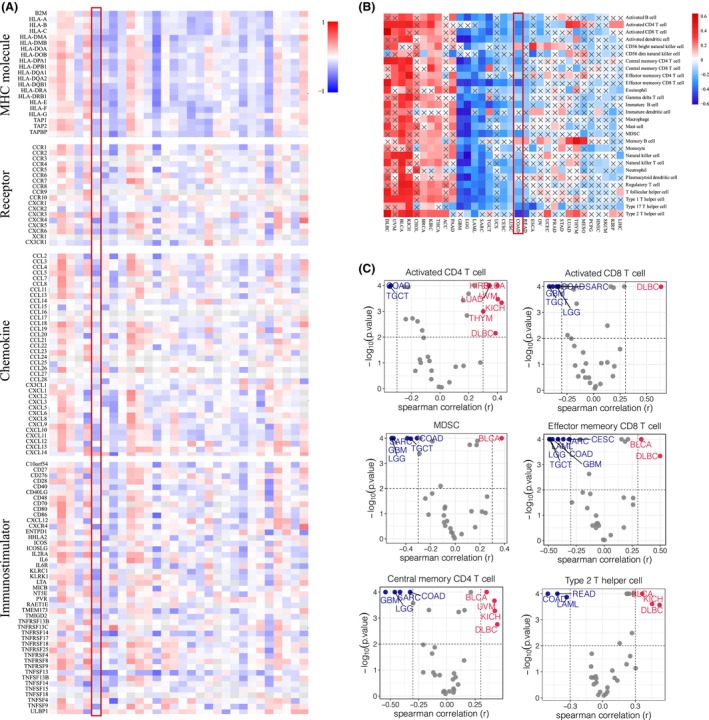
Pan‐cancer immunological correlation of GNG4. (A) Correlation between *GNG4* expression and pan‐cancer immunomodulators, including MHC molecules, receptors, chemokines and immunostimulators. (B) Correlation between *GNG4* expression and immune cells across cancer types. (C) *GNG4* expression was negatively correlated with the majority of immune cells in CC.

**FIGURE 5:**
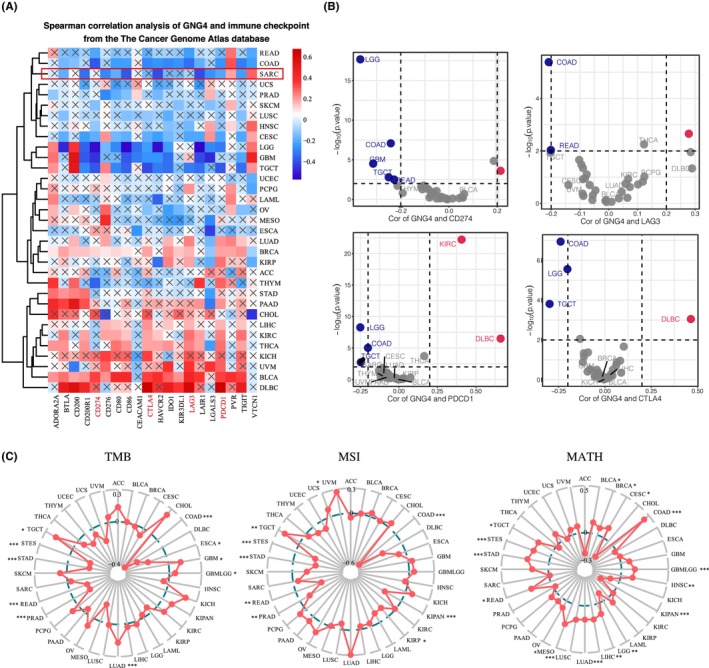
Correlation between *GNG4* expression and TMB, MSI and MASH across cancer types. (A) Correlation between *GNG4* expression and immune checkpoints across cancer types. (B) GNG4 was negatively associated with immune checkpoints, including CD274 (PDL1), LGA3, CTLA‐4 and PDCD1 (PD1) in CC. (C) Correlation between *GNG4* expression and TMB, MSI and MASH.

Pan‐cancer analyses aim to depict the immunological role of GNG4 comprehensively and thus to determine cancer types that may benefit from anti‐GNG4 immunotherapy. Just as GNG4 was expressed inconsistently in tumours, the relationship between GNG4 and immunomodulators (Figure 4A) or immunoregulatory cells was diverse (Figure 4B). Of note, *GNG4* expression was negatively correlated with a majority of immunomodulators, including MHC molecules, receptors, chemokines and immunostimulators in CC. Similarly, there was a significant negative correlation between *GNG4* expression and TIICs in the tumour microenvironment (TME) that were observed by using the ‘ssGSEA’ algorithm (Figure 4B). Among them, the immune cells negatively correlated with GNG4 included activated CD4^+^ T cells, CD8^+^ T cells, MDSC, effector memory CD8^+^ T cells, central memory CD4^+^ T cells and type 2 T helper cells (Figure 4C). Furthermore, we evaluated the correlation between *GNG4* expression and immune checkpoints in multiple cancers and demonstrated that GNG4 was negatively associated with immune checkpoints, including CD274 (PDL1), LGA3, CTLA‐4 and PDCD1 (PD1) in the COAD data set (Figure 5A,B). The TMB and MSI status were found to be potential determinants of response and resistance to ICBs. Thus, we analysed the relevance between *GNG4* expression and TMB, MSI and MASH. We noted that there was a strong negative correlation with TMB and MSI in CC and a significant positive correlation with MASH. This result implied the potential mechanisms of how GNG4 functions in tumour progression and ICB therapy (Figure 5C, Figure S2).

In summary, pan‐cancer analysis suggested that *GNG4* is an oncogene. Patients with high *GNG4* expression have a higher tumour stage and poorer prognosis. The immune relationships of *GNG4* expression in the TME were diverse, indicating that the potential regulation mechanisms by GNG4 differ. In CC, GNG4 had an obvious immunosuppressive effect, suggesting the potency of CC for anti‐GNG4 immunotherapy.

### 3.5 Expression, DNA methylation and mutational analyses of *GNG4* in CC

**FIGURE 6:**
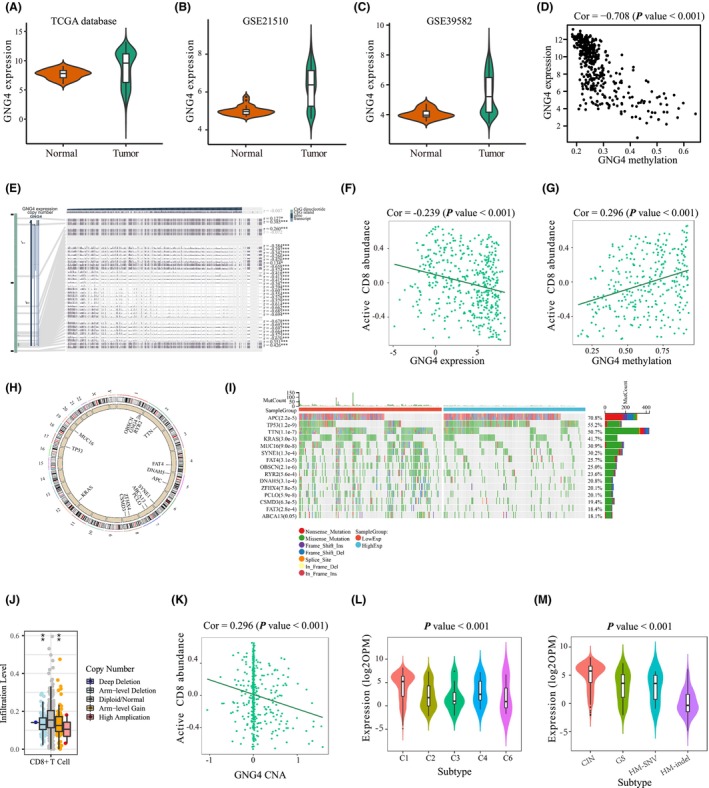
Expression, DNA methylation and mutational analyses of *GNG4* in CC. (A–C) *GNG4* expression between tumour and adjacent normal tissue in TCGA (A), GSE39582 (B) and GSE21510 (C) data sets. (D) Significantly negative correlation between *GNG4* expression and methylation in the TCGA database. (E) Correlation between CpG island methylation and *GNG4* expression in the MEXPRESS database. (F, G) CD8^+^ T cells were negatively associated with *GNG4* expression (F), while positively associated with *GNG4* methylation (G). (H) *GNG4* CNV on chromosomes. (I) Heat map of the 15 most frequently mutated genes in CC and their overall mutation rates were higher with low expression of *GNG4*. (J) The level of CD8^+^ T cells varied with changes of *GNG4* CNVs. (K) The *GNG4* CNV was negatively correlated with CD8^+^ T cells in the TISIDB database. (L, M) *GNG4* expression varied with immune subtypes (L) and molecular subtypes (M) in CC in the TISIDB database.

The extent of malignancy associated with GNG4 in CC was further assessed in the TCGA, GSE39582 and GSE21510 data sets. *GNG4* was stably and highly expressed in cancer tissues compared with normal adjacent tissues in the TCGA and GSE39582 data sets, as discussed previously (Figure 6A,B). A similar result was obtained from GSE21510, which had 123 tumour and 25 normal tissues (Figure 6C). As is well known, DNA methylation is crucial in tumorigenesis. In addition, we found that GNG4 expression was lower in poorly differentiated colon cancer tissues compared with moderately/well differentiated colon cancer tissues (Figure S1A).

The *GNG4* methylation level was significantly lower in cancer tissues compared with normal tissues (Figure S1B). As demonstrated, the *GNG4* methylation level was significantly negatively correlated to expression levels in the TCGA database (COR = −0.708, *p* < 0.001, Figure 6D). Further analysis of the MEXPRESS database revealed a strong negative correlation between *GNG4* expression and CpG island methylation (Figure 6E). In addition, the abundance of CD8^+^ T cells in the TME was negatively correlated with *GNG4* expression (Figure 6F), and positively correlated with *GNG4* methylation (Figure 6G), suggesting that the immunosuppressive role of *GNG4* overexpression may be regulated by methylation. The molecular subtype can also be used for selecting neoadjuvant chemotherapy, radiotherapy and several targeted therapies by predicting the clinical response. The position of the *GNG4* CNV on the chromosomes was plotted by the TCGA database (Figure 6H). As shown in Figure 6F, the 15 most commonly mutated genes in CC had higher mutation rates in the low‐expression *GNG4* group (Figure 6I). In addition, the level of CD8 ^+^ T cells varied with changes of the *GNG4* CNV (Figure 6J). In the TISIDB database, the *GNG4* CNV also showed a negative correlation with CD8^+^ T cells (Figure 6K).

*GNG4* expression also varied with different immune subtypes and molecular subtypes in CC. Immune subtype C6 (TGF‐β dominant) patients had the lowest *GNG4* expression, while immune subtype C1 (wound healing) patients had the highest *GNG4* expression (Figure 6L). Patients with molecular subtypes CIN were more likely to have the lowest *GNG4* expression, while immune subtype C1 (wound healing) patients had the highest *GNG4* expression. Patients with molecular subtypes CIN were more likely to have relatively high levels of *GNG4*, while molecular subtypes HM‐indel patients had the lowest *GNG4* expression levels (Figure 6M).

### 3.6 *GNG4* promotes the proliferation, migration and invasion of CC cells

**FIGURE 7:**
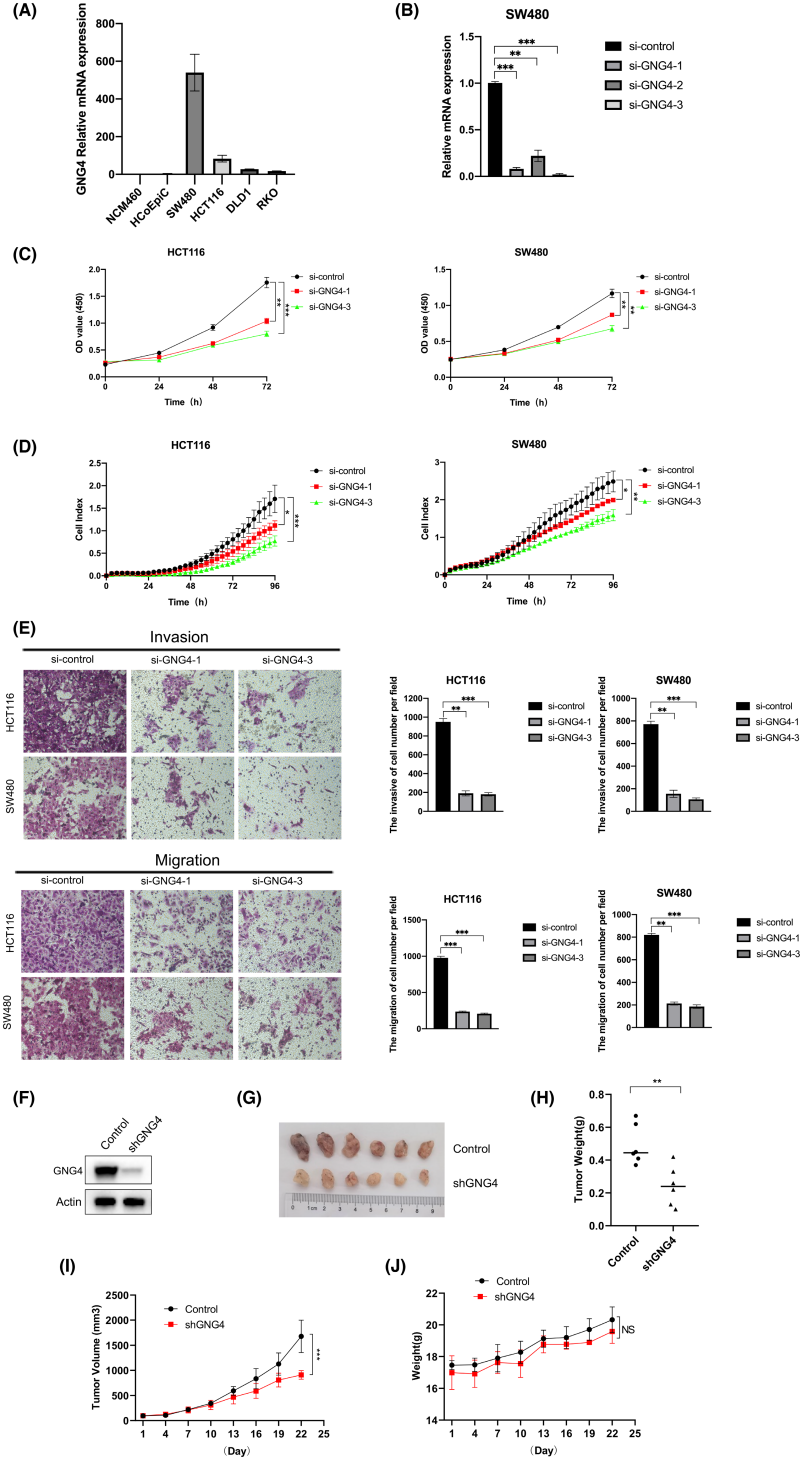
The target cell line was established and GNG4 induced proliferation, migration and invasion. (A) Quantitative PCR (qPCR) analysis of *GNG4* basal mRNA expression in six CC cell lines. *GNG4* mRNA expression levels were normalized according to the *GAPDH* expression level. (B) qPCR analysis of the efficiency of knocking down *GNG4* in SW480 cells. (C) Viability curves of *GNG4* knockdown in SW480 cells and HCT116 and SW480 cells for 0, 24 h, 48 h, 72 h and 96 h. (D) Knockdown of *GNG4* in SW480 and HCT116 cells were assessed for viability using RTCA. (E) Comparison of the migration and invasion of SW480 and HCT116 cells using Transwell compartments. (F) *GNG4* was knocked down in CC cell lines HCT116 using shRNA. The protein levels of GNG4 were analysed by Western blotting. (G) Photographic images of xenograft tumours from control and sh*GNG4* groups. Tumour‐bearing mice were randomly assigned to different groups (Randomized block design). (H) Pictures showed tumour weight both in control and sh*GNG4* groups. (I) Tumour volume were measured every 3 days for up to 22 days. (J) Mouse body weight were recorded every 3 days for up to 22 days. The bars represent the mean ± SD from three independent experiments. *p < 0.05; **p < 0.01; ***p < 0.001.

The relative expression of *GNG4* on the mRNA level (Figure 7A) in human CC cells SW480, HCT116, DLD1 and RKO, and human colon epithelial cells NCM460 and HCoEpiC was evaluated.

The level of *GNG4* was high in SW480 and HCT116 cells, moderate in DLD1 and RKO cells and lower in NCM460 and HCoEpiC cells. Next, we established *GNG4* knockdown SW480 cells (Figure 7B). The successful modulation of the *GNG4* expression level in SW480 cells was confirmed via qPCR. Then the *GNG4* knockdown SW480 cells and HCT116 cells were incubated for 0, 24, 48, 72 and 96 h (5% CO_2_ at 37°C, Figure 7C,D). The outcome of the CCK‐8 and RTCA assays demonstrated that GNG4 accelerated the growth of CC cells. In addition, in vitro analysis of migration and invasion was employed using the SW480 and HCT116 cells, which illustrated that *GNG4* knockdown promoted migration and invasion of SW480 and HCT116 cells (Figure 7E). To further investigate the role of *GNG4* in CC, we knocked down endogenous *GNG4* in HCT116 cells (Figure 7F). To further verify the effects in vivo, 12 nude mice bearing HCT116 tumours were randomly assigned into two groups: control and sh*GNG4*. Tumour volume and weight were dramatically decreased in sh*GNG4* groups (Figure 7G–I). There was no significant difference in average body weight between the control and sh*GNG4* groups (Figure 7J). Collectively, these findings suggested that GNG4 was an agonist of CC cell proliferation, migration and invasion.

### 3.7 *GNG4* expression and the immune score and immune cells in CC

**FIGURE 8:**
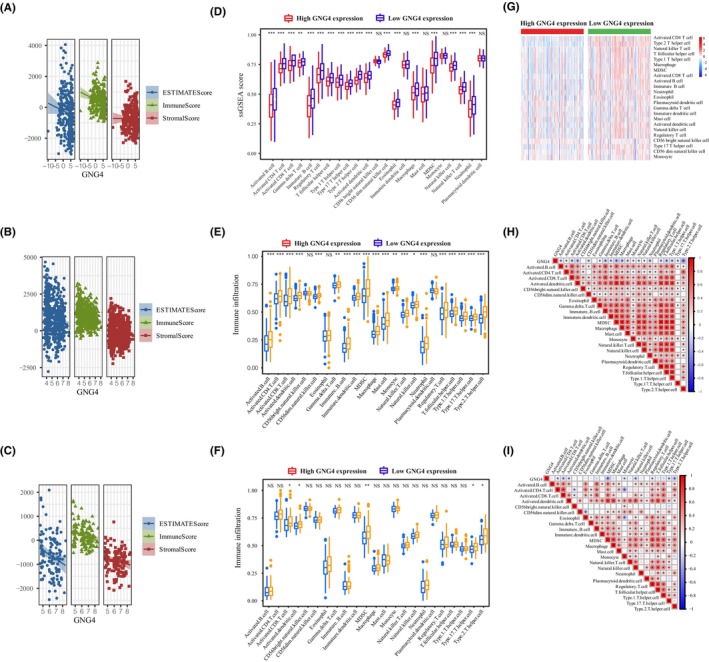
*GNG4* expression and immune cells and genetic heterogeneity in CC. (A–C) The relationship between *GNG4* expression and stromal score, immune score and estimate score in the TCGA database (A), GSE39582 (B) and GSE21510 (C). (D–F) Expression of immune cells in the high expression *GNG4* group and low expression *GNG4* group in the TCGA database (D), GSE39582 (E) and GSE21510 (F). (G–I) *GNG4* expression was negatively correlated with a variety of immune cells in the TCGA database (G), and GSE39582 (H) and GSE21510 (I) data sets.

We explored the association of *GNG4* with the immune score of CCs in the TCGA database and GSE38582 and GSE21510 data sets. The results indicated that there were negative correlations between *GNG4* expression and the immune score, but not the stromal score or estimate score in the TCGA (Figure 8A), GSE39582 (Figure 8B) or GSE21510 (Figure 8C) data sets, further illustrating the specificity of GNG4. Next, we explored whether GNG4 also affected the expression levels of other cells in the TME. The activity of immune cells in the high *GNG4* expression group decreased, including activated CD4^+^ T cells and CD8^+^ T cells, macrophages, Th1 cells, NK cells, DCsl and Th17 cells, leading to reduced TME infiltration, which promoted the growth of the tumour (Figure 8D–F). Furthermore, *GNG4* expression was negatively correlated with a variety of immune cells in the TCGA database (Figure 8G, Figure S3), GSE39582 (Figure 8H) and GSE21510 (Figure 8I).

### 3.8 *GNG4* expression and immune checkpointing in CC

**FIGURE 9:**
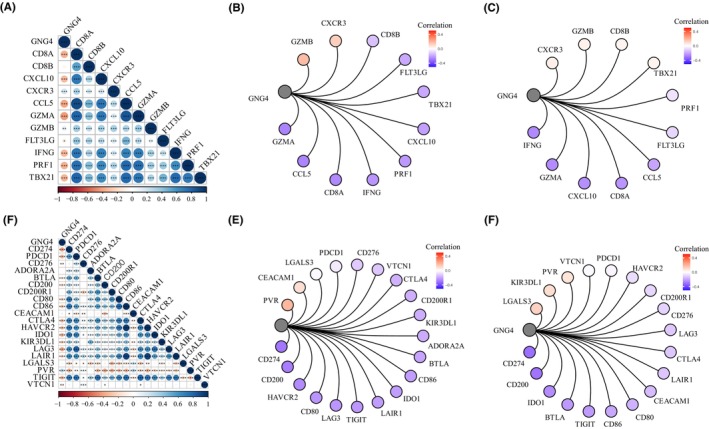
*GNG4* expression and immune checkpointing in CC. (A–C) The association of GNG4 with chemokines was evaluated in the TCGA database (A) and validated in the GSE39582 (B) and GSE1510 data sets (C). (D–F) The association of *GNG4* with chemokine receptors was evaluated in the TCGA database (D) and validated in the GSE39582 (E) and GSE1510 data sets (F).

Given that chemokines and chemokine receptors can recruit immune cells, including CD8^+^ T cells into the TME, we evaluated the association of *GNG4* with these factors in the TCGA database and validated the factors in the GSE39582 and GSE1510 data sets. The result showed that *GNG4* expression was highly negatively correlated with chemokines such as GZMA, CCL5, IFNG and CD8A (Figure 9A–C) and the chemokine receptors CD274, CD200, CD80 and TIGIT (Figure 9D–F). These results revealed that GNG4 could be a potential immunomarker and therapeutic target by regulating T cells in the TME of CC.

### 3.9 *GNG4* predicts clinical benefit of ICB

**FIGURE 10:**
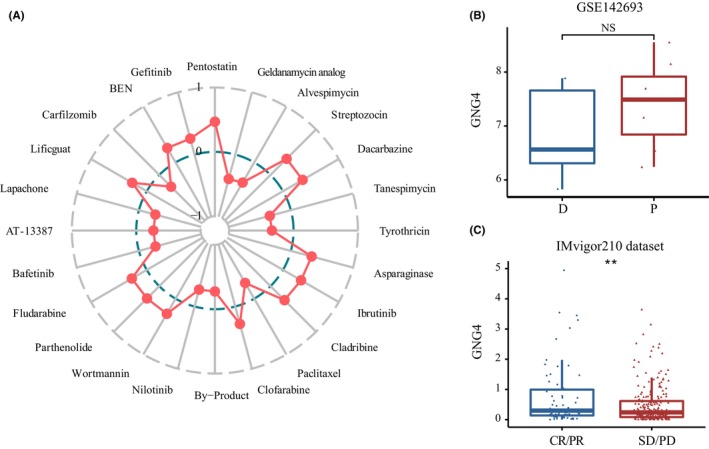
GNG4 predicts the clinical benefit of ICB in CC (A) Twenty‐four antitumor drugs were associated with *GNG4* expression in the CellMiner web tool. (B, C) The diagnostic value of *GNG4* expression for immunotherapy in GSE142693 (B) and validated in the IMvigor210 data set (C).

The correlation between *GNG4* expression and drug responses were revealed by the CellMiner (https://discover.nci.nih.gov/cellminer/) web tool. Patients with high expression of *GNG4* benefited from targeted drugs such as pentostatin, streptozocin and dacarbazine, while they had poor responses to a geldanamycin analog and alvespimycin (Figure 10A and Figure S4). We then investigated whether *GNG4* expression could predict an immunotherapy benefit using the GSE142693 data set and validating with the IMvigor210 data set. There was no statistically significant difference in *GNG4* expression to immunotherapy in GSE142693, possibly due to the small sample size (Figure 10B). However, in the IMvigor210 data set, the results showed that patients with low *GNG4* expression subtypes responded better to PD1 anti‐PD‐L1 therapy, whereas patients with high *GNG4* subtypes had CR or PR (Figure 10C). These results suggested that GNG4 can predict the effects of ICB treatment.

## 4 DISCUSSION

Immune infiltration is closely associated with the pathogenesis and progression of CC. TIICs, affecting angiogenesis and metastasis of colon cancer (mCC), are appealing therapeutic targets.^24^ Recently, the ICBs for CC, including ICIs and CAR‐T cells, have been less effective for patients, which was caused by the low immune cell infiltration level into the TME and by effector T cell exhaustion.^25^ The mCC patients with dMMR or MSI‐H receive a clear clinical responses, while those with proficient MMR or microsatellite stable tumours did not benefit from immunotherapy.^7^ Hence, defining the molecular mechanisms and development of predictive biomarkers for immunotherapy strategies are urgently required.

For mechanism studies of CC progression and the response to immunotherapy interventions, aberrantly expressed genes associated with an immune score were screened by WGCNA, and *GNG4* was subsequently selected through prognostic and immune correlation analysis. Our study revealed the potential value of *GNG4* as predictor of prognosis and clinical response to ICB.

Findings from preclinical research^26^ showed that GNG4 could be used as a potential biomarker to predict the response of immunotherapy in bladder cancer, implying that GNG4 may be a broad‐spectrum therapeutic target. Hence, we conducted a comprehensive pan‐cancer evaluation. *GNG4* mRNA levels were highly expressed and positively correlated with various tumour stages, including LUAD, PAAD, COAD, KIRP, BLCA and LIHC, implying its potential carcinogenic effect. *GNG4* expression was positively correlated with TNM stages, suggesting that GNG4 may be involved in tumour metastasis and progression. The high expression of *GNG4* predicted shorter survival in COAD and LUAD, which was consistent with previous studies.^16^, ^17^, ^27^ A pan‐cancer immunological correlation of GNG4 aimed to determine cancer types that may benefit from anti‐GNG4 immunotherapy. The results showed that the relationship between GNG4 and immunomodulators or immunoregulatory cells was diverse (Figure 2A,B), indicating the heterogeneity of *GNG4* regulation. Of note, *GNG4* expression was negatively correlated with a majority of immunomodulators, including MHC molecules, receptors, chemokines and immunostimulators in CC. Finally, CC was shown to be an ideal cancer for anti‐GNG4 immunotherapy.

Despite the clinical success of antibodies against immunomodulators such as PD‐L1/PD‐1 and CTLA4, only a small fraction of individuals have a lasting benefit, suggesting the urgency of an efficient cancer‐immunity cycle to iteratively proceed and expand, and thus generate anticancer immune responses.^28^, ^29^ Our research revealed that GNG4 may be involved in the initiation of several key steps in the cancer immune cycle. Tumour‐infiltrating lymphocytes in the TME have been shown to be efficient in predicting prognosis and immunotherapeutic efficacy for cancer.^30^ In the TCGA‐COAD cohorts, the infiltration levels of several effector TIICs, such as activated CD8^+^ T cells, activated CD4^+^ T cells, activated B cells, Type 1 T helper cells, macrophages and natural killer cells, were significantly downregulated in CC patients with high *GNG4* expression, which was verified in the validation group. Moreover, high *GNG4* expression was also promoted in the C1 (wound healing) immune subtypes and CIN molecular subtypes, which indicated that GNG4 may be involved in TME remodelling.

We found that the *GNG4* methylation status was closely related to its mRNA expression and positively correlated with CD8^+^ T cells. In addition, patients with a *GNG4* gene copy number amplification had lower CD8^+^ T cells. These results show that the amplification and methylation of *GNG4* may be a genetic and epigenetic event in CC, contributing to the remodelling of the immune microenvironment.

ICB targeting these ICPs, such as PD1‐blocking antibodies, pembrolizumab and nivolumab, have shown efficacy in patients with metastatic CRC that is dMMR‐MSI‐H, and they have been granted accelerated FDA approval.^5^ Our study showed a strong relationship between GNG4 and ICPs, such as PD‐L1, CD200, LAG‐3, CTLA4 and IDO1, which provides a theoretical basis for the development and application of certain drugs in the future.

TMB and MSI were shown to be clear biomarkers for a potential response to immunotherapy in colorectal and other solid tumours.^5^, ^31^ Zhao et al. demonstrated that GNG4 might play an important role in CRC TMB.^18^ GNG4 was negatively correlated with TMB and MSI in several cancers, with the strongest correlation detected in CC. These results indicated the unique role of GNG4 in CC immunogenicity.

GNG4 was shown to promote tumour progression in CRC^16^; however, its potential mechanisms remain unclear. As a result, increased GNG4 induced the proliferation, migration and invasion of CC cells. The GSEA analysis revealed a marked enrichment of MYC targets, E2F targets, the inflammatory response and DNA repair in the high *GNG4* expression group. The MYC oncogene is a grand orchestrator of cancer growth and immune evasion, and it can regulate the TME by affecting both innate and adaptive immune effector cells and immune regulatory cytokines.^32^, ^33^ Relating to immune infiltration, E2Fs are potential biomarkers for prognosis of many cancers, including human gastric carcinoma, pancreatic adenocarcinoma and clear cell renal cell carcinoma.^24^, ^25^, ^26^, ^27^, ^28^, ^29^, ^30^, ^31^, ^32^, ^33^, ^34^, ^35^, ^36^ In addition, low expression of *GNG4* was associated with enrichment in the TNF‐γ and INF‐α signalling pathways. INF‐α belongs to Type I IFNs and has been reported to be associated with immune‐mediated and inflammatory disorders.^37^ IFN‐γ is mainly produced by natural killer (NK) and T helper cells and involved in inflammation and autoimmunity.^38^ In summary, the invasion and metastasis of CC was promoted by GNG4 via multiple mechanisms, which may also explain the tumour immune escape.

Furthermore, we found that patients with a low *GNG4* expression subtype responded better to PD1 anti‐PD‐L1 therapy, suggesting that *GNG4* expression can predict ICB treatment efficacy, based on the IMvigor210 data set. Although more studies are still needed for further confirmation, the immune microenvironment and the immunotherapy response of CC may be related to GNG4.

To conclude, we comprehensively analysed correlations between immune infiltration and oncogenes in the TME of CC. *GNG4* was screened and found to affect the invasion and migration of CC cells and regulate the immune microenvironment remodelling in CC. GNG4 has an obvious immunosuppressive effect that indicated the crucial roles of CC for anti‐GNG4 immunotherapy.

## AUTHOR CONTRIBUTIONS

**Juan Wang:** Conceptualization (equal); data curation (equal); writing – review and editing (equal). **Yanshuang Wang:** Data curation (equal). **Jiaming Zhou:** Data curation (equal). **Mengmeng Cai:** Formal analysis (equal). **Peng Guo:** Writing – review and editing (equal). **Tongde Du:** Funding acquisition (equal); writing – review and editing (equal). **Hui Zhang:** Writing – original draft (equal); writing – review and editing (equal).

## CONFLICT OF INTEREST STATEMENT

The author declared no competing interests.

## Supporting information


Figure S1

Figure S2

Figure S3

Figure S4

## Data Availability

The data used to support the findings of this study are available from the corresponding author upon request.
