# Supplementary figures and images for: GNG4, as a potential predictor of prognosis, is correlated with immune infiltrates in colon adenocarcinoma
Source: J Cell Mol Med. 2023 Jul 13;27(17):2517–32. doi: 10.1111/jcmm.17847 (PMC10468912; doi:10.1111/jcmm.17847)

A

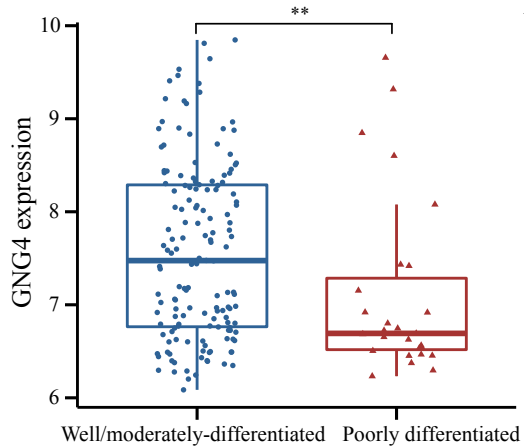

B

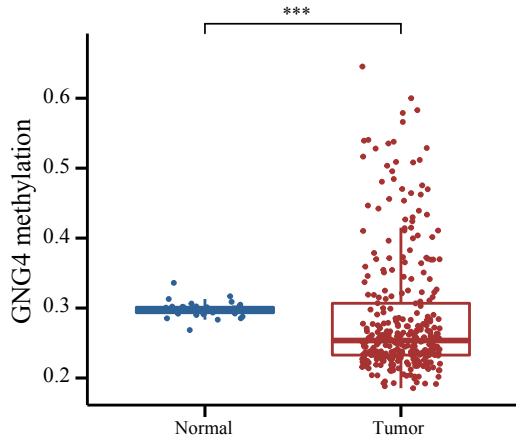

Supplement: Supplementary file 1 — Figure S1 [file JCMM-27-2517-s001.pdf]

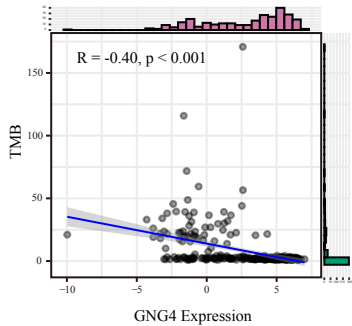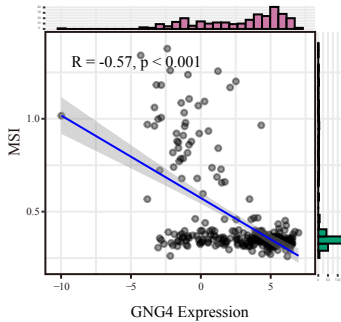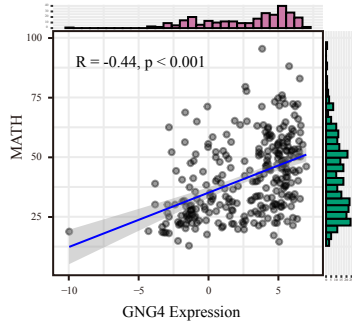

Supplement: Supplementary file 2 — Figure S2 [file JCMM-27-2517-s004.pdf]

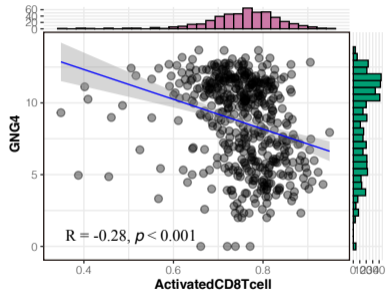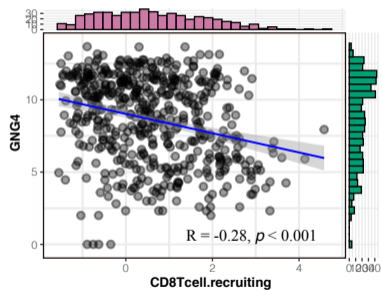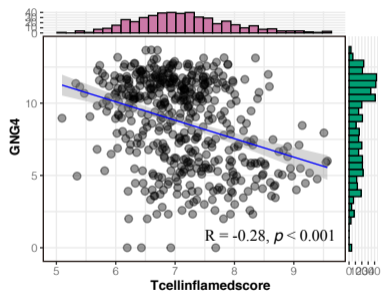

Supplement: Supplementary file 3 — Figure S3 [file JCMM-27-2517-s003.pdf]
