# Supplementary material for: GNG4, as a potential predictor of prognosis, is correlated with immune infiltrates in colon adenocarcinoma
Source: J Cell Mol Med. 2023 Jul 13;27(17):2517–32. doi: 10.1111/jcmm.17847 (PMC10468912; doi:10.1111/jcmm.17847)
Supplement: Supplementary file 4 — Figure S4 [file JCMM-27-2517-s002.pdf]

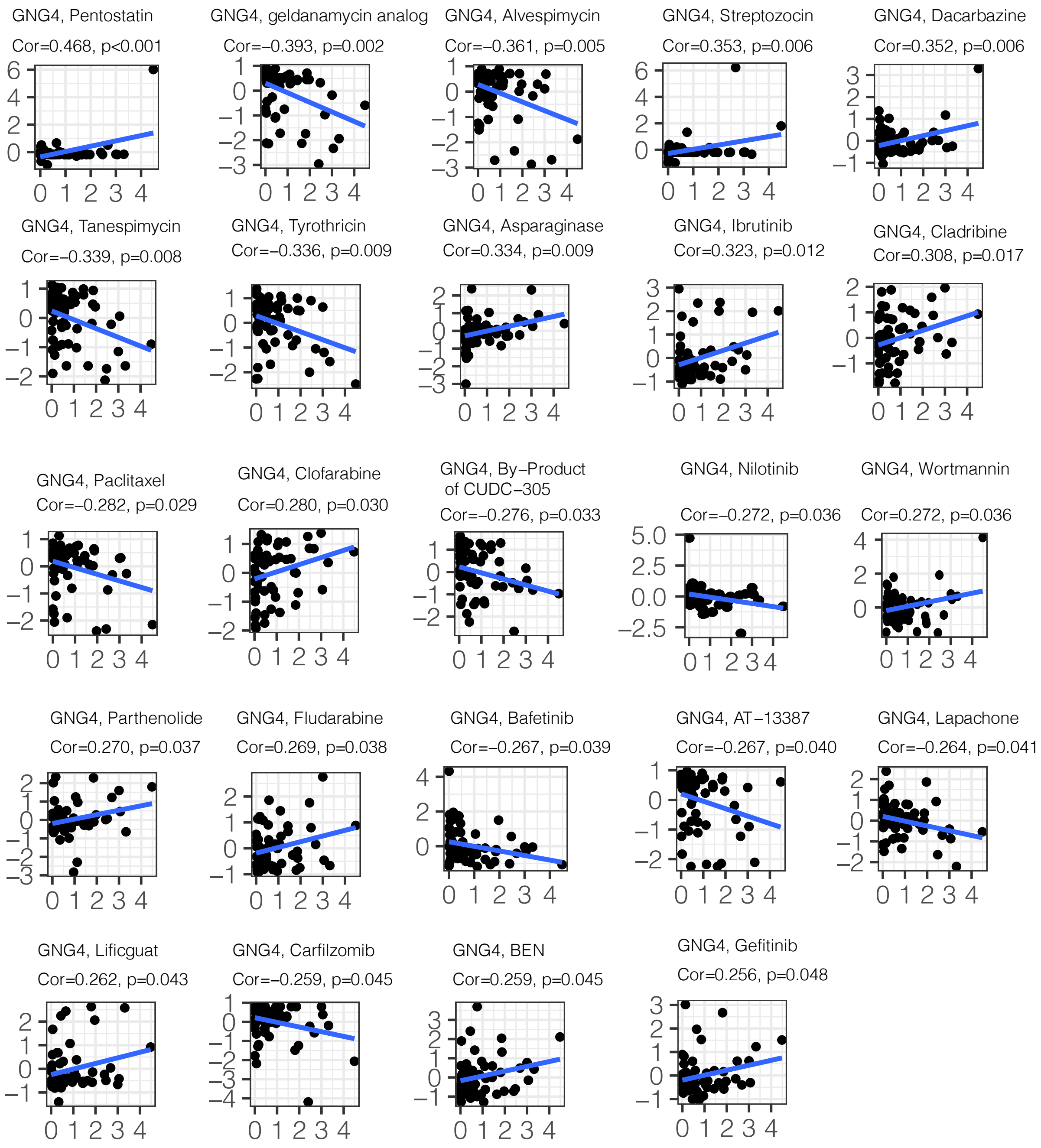
GNG4, Pentostatin
Cor=0.468, p<0.001
GNG4, geldanamycin analog
Cor=−0.393, p=0.002
GNG4, Alvespimycin
Cor=−0.361, p=0.005
GNG4, Streptozocin
Cor=0.353, p=0.006
GNG4, Dacarbazine
Cor=0.352, p=0.006
GNG4, Tanespimycin
Cor=−0.339, p=0.008
GNG4, Tyrothricin
Cor=−0.336, p=0.009
GNG4, Asparaginase
Cor=0.334, p=0.009
GNG4, Ibrutinib
Cor=0.323, p=0.012
GNG4, Cladribine
Cor=0.308, p=0.017
GNG4, Paclitaxel
Cor=−0.282, p=0.029
GNG4, Clofarabine
Cor=0.280, p=0.030
GNG4, By−Product of CUDC−305
Cor=−0.276, p=0.033
GNG4, Nilotinib
Cor=−0.272, p=0.036
GNG4, Wortmannin
Cor=0.272, p=0.036
GNG4, Parthenolide
Cor=0.270, p=0.037
GNG4, Fludarabine
Cor=0.269, p=0.038
GNG4, Bafetinib
Cor=−0.267, p=0.039
GNG4, AT−13387
Cor=−0.267, p=0.040
GNG4, Lapachone
Cor=−0.264, p=0.041
GNG4, Lificguat
Cor=0.262, p=0.043
GNG4, Carfilzomib
Cor=−0.259, p=0.045
GNG4, BEN
Cor=0.259, p=0.045
GNG4, Gefitinib
Cor=0.256, p=0.048
